# Community Awareness and Knowledge About Precocious Puberty in Riyadh City, Saudi Arabia

**DOI:** 10.7759/cureus.70347

**Published:** 2024-09-27

**Authors:** Sarah S AlSubaie, Rewaida R Bin Muaibed, Tala S AlManea, Sara K Alshaibani, Norah S Al Towaim, Lama A Alahmari, Maha M Alanazi, Ahmed M Almutairi

**Affiliations:** 1 College of Medicine, Imam Mohammad Ibn Saud Islamic University (IMSIU), Riyadh, SAU; 2 Department of pediatrics, College of Medicine, Imam Mohammad Ibn Saud Islamic University (IMSIU), Riyadh, SAU

**Keywords:** precocious puberty, precocious puberty community awareness, precocious puberty complications awareness, precocious puberty in riyadh city, precocious puberty in saudi arabia

## Abstract

Background

Puberty is the process of physical maturation where an adolescent reaches sexual maturity and becomes capable of reproduction. On average, puberty typically begins between 8 and 13 years old in females and 9 and 14 years old in males. The traditional definition of precocious puberty is the development of secondary sexual characteristics before eight years of age in girls and nine years in boys. In this study, we aimed to assess the knowledge and awareness of Riyadh residents in Saudi Arabia about precocious puberty and its complications.

Methodology

A cross-sectional study was conducted to estimate the awareness and knowledge among Riyadh residents about precocious puberty and its complications; 426 participants were included in this study. Participants completed a pretested self-administered questionnaire that included sociodemographic data, knowledge about precocious puberty definition, risk factors, complications, and treatment.

Results

This study enrolled 426 participants. Overall, 10.1% were correct about the precocious puberty age for girls, and 8.5% knew about the precocious puberty age for boys. The overall mean knowledge score among the study sample was 5.56 (SD = 2.79), with poor knowledge being prominent and constituting 68.8%, while fair and good knowledge were 30% and 1.2%, respectively. Working in a non-medical field was the sole independent significant predictor of incorrect answers of knowledge about precocious puberty.

Conclusions

Awareness strategies are needed to increase awareness among the community to encourage prevention and treatment for precocious puberty as the awareness and knowledge of Riyadh residents in Saudi Arabia about precocious puberty and its complications are low.

## Introduction

Puberty is the process of physical maturation, where an adolescent reaches sexual maturity and becomes capable of reproduction. On average, puberty typically begins between 8 and 13 years old in females and 9 and 14 in males [1]. Most boys’ and girls’ pubertal development follows a predictable pattern. The main hormone of the reproductive endocrine system is gonadotropin-releasing hormone (GnRH), which regulates the production of female luteinizing hormone (LH) and follicle-stimulating hormone (FSH) from pituitary gonadotropic cells [1]. An increase in the pulsatile release of GnRH from the hypothalamus is considered a critical hormonal event throughout puberty. The activation of steroidogenesis and gametogenesis from the gonads by FSH and LH results in the development of secondary sexual characteristics and fertility, such as pubic hair development (pubarche), genital changes in boys, voice changes, an increase in height, and the development of breasts in girls (thelarche) along with the onset of menstruation (menarche). Puberty proceeds through five stages, termed Tanner stages, ranging from prepubertal to full maturity [1,2].

The traditional definition of precocious puberty is the development of secondary sexual characteristics before eight years of age in girls and nine years in boys [3]. However, environmental (secular trends, adoption, single parenthood, and potential exposure to estrogenic endocrine-disrupting chemicals) and dietary (body mass index) variables, as well as constitutional (genetics, ethnicity) factors, may influence the timing of puberty [4]. The gradual nature of the puberty transition can be utilized to explain the diversity of precocious puberty in terms of clinical presentation and characterization [5]. The primary responsibility for early screening lies with the parents, who serve as the initial line of defense in identifying potential health issues in their children. As a result, parents must monitor their children constantly to recognize signs of precocious puberty early and seek suitable therapies, ultimately enhancing the child’s overall well-being [6]. According to previous research studies, additional evaluation of parents’ knowledge of precocious puberty is strongly recommended. In this research, our objective was to evaluate the knowledge and awareness of precocious puberty and its associated complications among residents in Riyadh, Saudi Arabia.

## Materials and methods

Study Design and Settings

This observational, cross-sectional study aimed to evaluate the awareness and knowledge of precocious puberty and its complications among Riyadh residents using a pretested questionnaire. Developed by a team of pediatricians, the questionnaire was reviewed and refined with their permission before being used in our research. The refined questionnaire was then distributed to a public sample for validation, and data were collected over a six-month period, from October 2022 to March 2023.

Study samples

Convenience sampling was used to choose the sample population, and 426 residents of Riyadh who met the following requirements were included in the study: Saudi Arabian and aged 18 years and above. Social media platforms were used to recruit participants, and online self-administered questionnaires were used to collect data and obtain participants’ consent to participate in the study.

Sample size

According to the data provided by the Saudi General Authority for Statistics, the population of Saudi adults (aged 20 years and above) residing in Riyadh in 2022 was 5,546,465. To ensure the accuracy of the findings, it was necessary to maintain an acceptable margin of error, which was set at 5%, alongside a design effect of 2, with a confidence level of 95%. Consequently, a minimum sample size of 385 was determined. In the context of this study, the total number of responses collected for analysis amounted to 426. The calculation of the sample size was conducted using Raosoft.

Data collection and management

The questionnaire was designed by a group of pediatric consultants and endocrinologists and was pre-tested by sending it to a group of people from the public to validate the questionnaire. The questionnaire included four sections. Sixteen criteria were used to identify the sociodemographic factors of the participants. The second section included three questions to assess the knowledge of precocious puberty definition and the age to diagnose precocious puberty, and each correct answer in this section was scored 2 points. The third section included 11 questions to assess awareness of precocious puberty risk factors, and each correct answer in this section was scored 1 point. Three questions were included in the last section to assess knowledge of precocious puberty complications and treatment of precocious puberty, and each correct answer in this section was scored 1 point. Participants who scored 14-20 points were considered as having good knowledge, 7-13 as fair knowledge, and 0-6 as poor knowledge. Personal information was not obtained to protect participants’ privacy, and participants were assured that this survey was intended for academic purposes only.

Statistical analysis

Categorical variables were described using counts and proportions (%), while means and standard deviations were used to present continuous variables. The relationship between the level of knowledge and the sociodemographic characteristics of participants was assessed using the chi-square test. Based on the significant results, a multivariate analysis was performed to determine the significant independent predictors of poor knowledge. A p-value of less than 0.05 was considered statistically significant. All statistical data were analyzed using SPSS version 26 (IBM Corp., Armonk, NY, USA).

## Results

This study enrolled 426 participants. As shown in Table 1, 25.4% of participants were between 18 and 29 years old, with females being dominant (86.4%). More than three-quarters were married (76.8%), and 73.5% had children. Respondents who were bachelor’s degree holders constituted 68.8%, while those who were employed were 33.3%. The proportion of participants who knew someone with precocious puberty was 23.2%, while those who were living with someone who had precocious puberty was 11.5%. Respondents who lived with someone who worked in the medical field were 33.3%, and those with a previous history of chronic illness were 17.4%.

**Table 1:** Sociodemographic characteristics of participants (n = 426).

Study variables	N (%)
Age group	
18–29 years	108 (25.4%)
30–39 years	101 (23.7%)
40–49 years	91 (21.4%)
50–59 years	100 (23.5%)
≥60 years	26 (06.1%)
Gender	
Male	58 (13.6%)
Female	368 (86.4%)
Marital status	
Married	327 (76.8%)
Unmarried	99 (23.2%)
Having children	
Yes	313 (73.5%)
No	113 (26.5%)
Number of children	
None	113 (26.5%)
One	14 (03.3%)
Two or more	299 (70.2%)
Educational level	
High school or below	74 (17.4%)
Diploma	10 (02.3%)
Bachelor degree	293 (68.8%)
Postgraduate	49 (11.5%)
Occupational status	
Student	64 (15.0%)
Employed	142 (33.3%)
Unemployed	128 (30.0%)
Retired	92 (21.6%)
Do you know someone who was diagnosed with precocious puberty?	
Yes	99 (23.2%)
No	327 (76.8%)
Are you living with someone who has precocious puberty?	
Yes	49 (11.5%)
No	377 (88.5%)
Do you work in the medical field?	
Yes	50 (11.7%)
No	376 (88.3%)
Do you live with someone who works in the medical field?	
Yes	142 (33.3%)
No	284 (66.7%)
Do you have a previous personal history of any chronic illness?	
Yes	74 (17.4%)
No	352 (82.6%)

As shown in Table 2, only 82.2% answered correctly about the meaning of precocious puberty. Also, nearly 10.1% were correct about the precocious puberty age for girls, and 8.5% knew about the precocious puberty age for boys. Furthermore, even though most of our respondents were correct about the statements “females normally reach pubertal age before males” (84.7%) and “hereditary factors play a role in developing precocious puberty” (77%), their knowledge was shallow on “Seasonal fruits might be a risk factor for developing precocious puberty?” (correct answer: 4.2%), “Preterm children have a higher chance to develop precocious puberty?” (correct answer: 4.2%) and “Lavender perfumes might be a risk factor for developing precocious puberty?” (correct answer: 3.3%). The overall mean knowledge score was 5.56 (SD = 2.79), and those with poor knowledge were the majority (68.8%).

**Table 2:** Assessment of knowledge about precocious puberty (n = 426).

Awareness statement	N (%)
Precocious puberty is child puberty before normal age. Do you agree with that? (yes)	350 (82.2%)
Precocious puberty for boys is before the child reaches the age of 9 and below)	65 (15.3%)
6	09 (2.1%)
7	09 (2.1%)
8	12 (2.8%)
9	36 (8.5%)
10	68 (16.0%)
11	37 (8.7%)
12	61 (14.3%)
13	33 (7.7%)
14	31 (7.3%)
15	27 (6.3%)
I don’t know	103 (24.2%)
Precocious puberty for girls is before the child reaches the age of 8 and below	350 (82.2%)
6	10 (2.3%)
7	12 (2.8%)
8	43 (10.1%)
9	108 (25.4%)
10	82 (19.2%)
11	30 (7.0%)
12	41 (9.6%)
13	17 (4.0%)
14	05 (1.2%)
15	08 (1.9%)
I don’t know	70 (16.4%)
Do you think that females normally reach pubertal age before males? (yes)	361 (84.7%)
Do you think that hereditary factors play a role in developing precocious puberty? (yes)	328 (77.0%)
Do you think that obese children have a higher risk of developing precocious puberty? (yes)	205 (48.1%)
Do you think that children with precocious puberty will have short stature in the future? (yes)	126 (29.6%)
Do you know what are the treatment options for precocious puberty? (medical and surgical)	110 (25.8%)
Do you think that some medications, such as anti-fungals and antihistamines, might be a risk factor for developing precocious puberty? (yes)	71 (16.7%)
Do you think that Black/African children have a higher risk of developing precocious puberty? (yes)	64 (15.0%)
Do you think that plastic products might be a risk factor for developing precocious puberty? (yes)	53 (12.4%)
Do you think that some cosmetics creams might be a risk factor for developing precocious puberty? (yes)	51 (12.0%)
Do you think that a nylon cover for a sandwich might be a risk factor for developing precocious puberty? (yes)	46 (10.8%)
Do you think that a female child diagnosed with precocious puberty will have a risk for breast cancer in the future? (yes)	46 (10.8%)
Do you think that seasonal fruits might be a risk factor for developing precocious puberty? (yes)	18 (4.2%)
Do you think that preterm children have a higher chance of developing precocious puberty? (yes)	18 (4.2%)
Do you think that lavender perfumes might be a risk factor for developing precocious puberty? (yes)	14 (3.3%)
Knowledge score (mean ± SD)	5.81 ± 2.99
Level of knowledge	
Poor	279 (65.5%)
Fair	140 (32.9%)
Good	07 (1.6%)

Table 3 shows the relationship between the level of knowledge and the sociodemographic characteristics of participants. It can be observed that the prevalence of participants who were correct or had fair answers of the precocious puberty definition was significantly more common among the younger age group (p < 0.001), employed/student (p < 0.001), and working in the medical field (p < 0.001). In contrast, the prevalence of those who had incorrect answers was significantly more common among married (p < 0.001) and those who were having children (p = 0.001).

**Table 3:** Relationship between the level of knowledge and the sociodemographic characteristics of participants (n = 426). §: P-value has been calculated using the chi-square test; **: Significant at p < 0.05 level.

Factor	Level of knowledge		P-value^§^
	Incorrect answer of definition N (%) (n = 279)	Correct or fair answer of definition N (%) (n = 147)	
Age group			
<40 years	119 (42.7%)	90 (61.2%)	<0.001**
≥40 years	160 (57.3%)	57 (38.8%)	
Gender			
Male	45 (16.1%)	13 (08.8%)	0.037**
Female	234 (83.9%)	134 (91.2%)	
Marital status			
Married	232 (83.2%)	95 (64.6%)	<0.001**
Unmarried	47 (16.8%)	52 (35.4%)	
Having children			
Yes	220 (78.9%)	93 (63.3%)	0.001**
No	59 (21.1%)	54 (36.7%)	
Educational level			
Diploma or below	61 (21.9%)	23 (15.6%)	0.125
Bachelor or higher	218 (78.1%)	124 (84.4%)	
Occupational status			
Unemployed	162 (58.1%)	58 (39.5%)	<0.001**
Employed/Student	117 (41.9%)	89 (60.5%)	
Do you know someone who was diagnosed with precocious puberty?			
Yes	60 (21.5%)	39 (26.5%)	0.243
No	219 (78.5%)	108 (73.5%)	
Are you living with someone who has precocious puberty?			
Yes	31 (11.1%)	18 (12.2%)	0.727
No	248 (88.9%)	129 (87.8%)	
Do you work in the medical field?			
Yes	16 (05.7%)	34 (23.1%)	<0.001**
No	263 (94.3%)	113 (76.9%)	
Do you live with someone who works in the medical field?			
Yes	93 (33.3%)	49 (33.3%)	1.000
No	186 (66.7%)	98 (66.7%)	
Do you have a previous personal history of any chronic illness?			
Yes	47 (16.8%)	27 (18.4%)	0.694
No	232 (83.2%)	120 (81.6%)	

In a multivariate regression model (Table 4), working in a non-medical field was the sole independent significant predictor of incorrect answers of knowledge about precocious puberty. This further indicates that compared to respondents who were working in a medical field, respondents who were working in a non-medical field were predicted to increase the chance of having incorrect answers about precocious puberty knowledge assessment by at least 3.5 times higher (adjusted odds ratio = 3.317; 95% confidence interval (CI) = 1.566-6.286; p = 0.001). Other sociodemographic variables included in the model did not show a significant effect after adjustment to regression estimates, including age, gender, having children, and marital status (p > 0.05).

**Table 4:** Multivariate regression analysis to determine the significant independent predictors associated with incorrect answers of knowledge about precocious puberty (n = 426). **: Significant at p < 0.05 level. AOR = adjusted odds ratio; CI = confidence interval

Factor	AOR	95% CI	P-value
Age group			
<40 years	Ref.		
≥40 years	1.343	0.813–2.218	0.250
Gender			
Male	Ref.		
Female	0.544	0.270–1.098	0.090
Marital status			
Married	Ref.		
Unmarried	0.341	0.087–1.341	0.123
Having children			
Yes	Ref.		
No	2.418	0.646–9.052	0.190
Occupational status			
Unemployed	Ref.		
Employed/Student	0.667	0.422–1.055	0.083
Do you work in the medical field?			
Yes	Ref.		
No	3.503	1.729–7.096	0.001 **

## Discussion

This study aimed to identify community awareness and knowledge about precocious puberty and its complications. Precocious puberty in children is one of the common endocrine diseases in pediatrics. The most consistent findings from studies of girls support the early timing hypothesis, which suggests that precocious girls have particularly difficult adjustment to puberty and are more likely to experience negative outcomes [7].

Epidemiological surveys have shown that the number of children with precocious puberty has significantly increased globally [8]. Precocious puberty negatively affects the physical and mental health of children and may increase the risk of hypertension, diabetes, and obesity [8]. Additionally, there could be a significant correlation in this modern age with larger weights of young Saudi females, with the consumption of fast foods related to early menarche and non-organic poultry consumption related to early thelarche [9]. Another study showed a result of a suggestive link between obesity and genetic factors that may contribute to delayed or advanced adulthood [10-12]. This highlights the need for greater community awareness.

Our findings revealed that participants who were younger, unmarried, and employed in the medical field had a better understanding of the condition. However, our multivariate regression analysis showed only those working in fields other than medicine had a poorer knowledge score, indicating a need for more knowledge dissemination on this condition among the general population. The findings of our study can catalyze medical professionals and health educators to enhance their comprehension of this condition. With a better understanding of precocious puberty, medical professionals can provide more effective care and treatment for those affected. The study also highlights the importance of educating the general population about this condition to ensure that parents and caregivers can recognize the signs of precocious puberty and seek medical help promptly.

Furthermore, it is important to note that pre-adolescents or adolescents experiencing precocious puberty often struggle with increased levels of anxiety, emotional sensitivity, and diminished self-esteem. A previous study reported that girls facing precocious puberty are more likely to struggle with heightened anxiety, intense emotions, body image concerns, and feelings of isolation compared to girls undergoing puberty at the expected age [13]. This data underscores the critical need to address and support the personal and social challenges associated with early puberty. Another study among teenage boys found that they need more information about health changes, mood swings, sexual orientation, and health behaviors during puberty [14]. Parents’ understanding of child development affects their expectations about their child’s growth and how they handle changes. Evidence shows that parents, especially mothers, have limited knowledge about factors that influence child development, such as obesity and the early signs of puberty [9,10].

Girls who show premature physical signs of puberty, significantly advanced bone age, below-expected height, and pubertal response to gonadotropin testing should be treated with GnRH agonists to arrest pubertal progression and improve adult height. The problem is that each of these variables is not yet well defined, and different investigators have different approaches [15].

The medical indications for precocious puberty are not all agreed upon. Treatment choices can vary depending on mental health conditions, height-related anxiety, and the nature of early menarche. Families and primary healthcare physicians should discuss the risks and make joint decisions about treatment options. The psychological impact of precocious puberty has not been fully investigated, but there are reports of miscarriage and early pregnancy. Psychiatric symptoms must be determined for each patient [11].

Therefore, raising public awareness of precocious puberty is essential. Parents should clearly know how to identify the early characteristics of sexual development, understand the adverse consequences of premature development, and seek medical attention for affected children as soon as possible. Treatment should be administered immediately after the diagnosis of precocious puberty [8].

Limitations

Our study, despite its limitations, provides significant insights. We acknowledge the use of convenience sampling, which may limit the generalizability of our findings. Additionally, the data may be skewed toward females, who often show more interest in childcare topics. However, these limitations do not diminish the value of our research, which can empower future studies in this field.

## Conclusions

Implementing awareness strategies that can significantly increase the community’s knowledge and understanding of precocious puberty in Saudi Arabia is crucial. This is particularly important as our paper showed that Riyadh residents’ awareness and knowledge of precocious puberty and its complications are currently low. By emphasizing the need for these strategies, we can make our audience, including medical professionals, educators, parents, and community members, feel the urgency and importance of their role in addressing this issue.
